# A Pre-mRNA-Splicing Factor Is Required for RNA-Directed DNA Methylation in *Arabidopsis*

**DOI:** 10.1371/journal.pgen.1003779

**Published:** 2013-09-12

**Authors:** Chao-Feng Huang, Daisuke Miki, Kai Tang, Hao-Ran Zhou, Zhimin Zheng, Wei Chen, Ze-Yang Ma, Lan Yang, Heng Zhang, Renyi Liu, Xin-Jian He, Jian-Kang Zhu

**Affiliations:** 1 Shanghai Center for Plant Stress Biology and Institute of Plant Physiology and Ecology, Shanghai Institutes for Biological Sciences, Chinese Academy of Sciences, Shanghai, China; 2 State Key Laboratory of Crop Genetics and Germplasm Enhancement, College of Resources and Environmental Science, Nanjing Agricultural University, Nanjing, China; 3 Department of Horticulture and Landscape Architecture, Purdue University, West Lafayette, Indiana, United States of America; 4 National Institute of Biological Sciences, Beijing, China; 5 Department of Botany and Plant Sciences, University of California, Riverside, California, United States of America; The University of Arizona, United States of America

## Abstract

Cytosine DNA methylation is a stable epigenetic mark that is frequently associated with the silencing of genes and transposable elements (TEs). In *Arabidopsis*, the establishment of DNA methylation is through the RNA-directed DNA methylation (RdDM) pathway. Here, we report the identification and characterization of RDM16, a new factor in the RdDM pathway. Mutation of *RDM16* reduced the DNA methylation levels and partially released the silencing of a reporter gene as well as some endogenous genomic loci in the DNA demethylase *ros1-1* mutant background. The *rdm16* mutant had morphological defects and was hypersensitive to salt stress and abscisic acid (ABA). Map-based cloning and complementation test led to the identification of *RDM16*, which encodes a pre-mRNA-splicing factor 3, a component of the U4/U6 snRNP. RNA-seq analysis showed that 308 intron retention events occurred in *rdm16*, confirming that RDM16 is involved in pre-mRNA splicing *in planta*. RNA-seq and mRNA expression analysis also revealed that the *RDM16* mutation did not affect the pre-mRNA splicing of known RdDM genes, suggesting that RDM16 might be directly involved in RdDM. Small RNA expression analysis on loci showing RDM16-dependent DNA methylation suggested that unlike the previously reported putative splicing factor mutants, *rdm16* did not affect small RNA levels; instead, the *rdm16* mutation caused a decrease in the levels of Pol V transcripts. ChIP assays revealed that RDM16 was enriched at some Pol V target loci. Our results suggest that RDM16 regulates DNA methylation through influencing Pol V transcript levels. Finally, our genome-wide DNA methylation analysis indicated that RDM16 regulates the overall methylation of TEs and gene-surrounding regions, and preferentially targets Pol IV-dependent DNA methylation loci and the ROS1 target loci. Our work thus contributes to the understanding of RdDM and its interactions with active DNA demethylation.

## Author Summary

Both plants and animals utilize cytosine DNA methylation as an important epigenetic mark to suppress transposable elements (TEs), repeat sequences and genes, which is crucial for the genome integrity and development. In plants, *de novo* DNA methylation can be mediated by the RNA-directed DNA methylation (RdDM) pathway. Plants have also evolved a pathway for active DNA demethylation that is initiated by the ROS1 subfamily of 5-methylcytosine DNA glycosylases, to counteract the RdDM pathway to prevent undesirable silencing. In this study, we identified RDM16, a new factor in the RdDM pathway. We show that RDM16 is a pre-mRNA splicing factor and its function in the regulation of DNA methylation and gene silencing is not through influencing siRNA levels or the expression or splicing of genes encoding known RdDM components, but likely through affecting Pol V transcripts. We also show that RDM16 preferentially affects ROS1 target loci. Together, our findings contribute to the understanding of RdDM and its interactions with ROS1-mediated DNA demethylation.

## Introduction

Cytosine methylation in eukaryotic cells is an epigenetic mark that plays important roles in diverse biological processes, such as the silencing of genes and transposons [1], [2], X inactivation [3], paramutation [4], and imprinting [5]. In plants, cytosine methylation can occur in all three sequence contexts: CG, CHG and CHH (H = A, T, or C). The *Arabidopsis* genome has 24% of CG, 6.7% of CHG and 1.7% of CHH sites methylated at the cytosine [6]. Maintenance of CG, CHG and CHH methylation is catalyzed by MET1, CMT3 and DRM2 enzymes, respectively [7]–[10]. Nevertheless, *de novo* cytosine methylation in all three sequence contexts can be catalyzed by DRM2 [10] in a pathway known as RNA-directed DNA methylation (RdDM) [11], [12]. In this pathway, a plant-specific RNA polymerase IV is recruited to transcribe transposons and some endogenous repeat loci and the transcripts are copied into double-stranded RNAs (dsRNAs) by RNA-DEPENDENT RNA POLYMERASE2 (RDR2) [13]–[16]. The dsRNAs are then processed into 24-nucleotide (nt) siRNA duplexes by DICER-LIKE 3 (DCL3) and the siRNAs were subsequently methylated at their 3′ ends by the RNA methylase HEN1 for stabilization [17], [18]. PolIV, RDR2, DCL3 and HEN1 are the key components for siRNA biogenesis and stability. In addition, the SNF2-like putative chromatin remodeling protein CLSY1 and the homeodomain transcription factor-like SHH1/DTF1, which interacts with Pol IV, assists in the Pol IV and RDR2-dependent siRNA biogenesis [19]–[21]. Following the methylation of the siRNA duplexes by HEN1, one strand of the siRNAs is loaded into AGO4 [22]. AGO4 interacts with the nascent transcript produced by Pol V, another plant-specific RNA polymerase, through base-pairing between the siRNA and nascent transcript [23]. Pol V transcription is facilitated by a complex formed by DRD1, DMS3 and RDM1 (termed DDR complex), and the transcripts serve as scaffolds for recruiting RdDM effector complex [24], [25]. AGO4 also interacts with the largest subunit NRPE1 of Pol V and KTF1, a homolog of yeast transcription elongation factor Spt5, to stabilize the association of AGO4 with the scaffold transcripts [26]–[28]. The RDM1 protein of DDR complex is associated with AGO4 and DRM2 and thus may help to recruit DRM2 to the region being transcribed by Pol V to catalyze DNA methylation [25], [29]. In addition to Pol V transcripts, Pol II-generated non-coding transcripts are also involved in the RdDM through recruiting the AGO4-containing effector complex [25], [30].

Besides AGO4, AGO6 and AGO9 are also involved in the RdDM, acting in a partially redundant manner with AGO4 [31], [32]. Recently, the DRM2 paralog DRM3 that is catalytically mutated was reported to play a role in the RdDM through promoting the activity of DRM2 [33]. More recently, a GHKL ATPase domain-containing protein DMS11 was identified as a new component of the RdDM machinery and was proposed to cooperate with DMS3 in the RdDM pathway by providing the missing ATPase function for DMS3 [34] or to regulate chromatin architecture [35]. Together, these results suggest that there is great complexity to the RdDM pathway and more components are likely required for modulating this important pathway. Moreover, the mechanisms through which Pol IV, Pol V and DRM2 are targeted to specific loci are still not fully understood.

In an attempt to identify genes involved in the RdDM, Ausin et al (2012) carried out a screen on T-DNA insertion lines by using *FWA* transgene silencing as a reporter system [36]. As a result, a splicing factor SR45 was identified and demonstrated to be required for the RdDM. Splicing factors are well known to be involved in the removal of introns from pre-mRNAs. In eukaryotic cells, pre-mRNA splicing takes place in a large multicomponent complex, called the splicesome, which is formed by ordered interactions of four small ribonucleoprotein particles (snRNPs), U1, U2, U4/U6 and U5 snRNPs, and numerous snRNP-associated proteins [37]. The U1 snRNP assembles with the 5′ splice site of pre-mRNA and recruits several splicing factors to form the commitment complex, and then the U2 snRNP interacts with the branch point of the introns to form the pre-spliceosome. Subsequently, the U5 snRNP associates with the U4/U6 snRNP to form a U4/U6.U5 tri-snRNP that assembles with the pre-spliceosome to form the mature spliceosome [37], [38]. *SR45* encodes a serine/arginine-rich (SR) protein belonging to a conserved family of structurally and functionally related non-snRNP proteins. SR45 is suggested to help with the formation of the bridge between 5′ and 3′ splice sites in the early stage of spliceosome assembly through interacting with U1-70K and U2AF^35^b proteins [39], [40]. The mechanism of SR45 function in RdDM is not understood. Although the abundance of Pol IV-dependent siRNAs is decreased in *sr45* mutant, it is still not known how SR45 is involved in the siRNA accumulation and it is possible that SR45 may play an indirect role through the splicing of genes that encode RdDM pathway components [36].

In this study, we report a U4/U6 snRNP associated protein RDM16, which is required for the RdDM. *RDM16* encodes a pre-mRNA-splicing factor 3 (PRP3) and is involved in the pre-mRNA splicing *in planta*. The *rdm16* mutation did not influence the pre-mRNA splicing of known RdDM genes, suggesting that RDM16 involvement in the RdDM might be direct. We also show that *rdm16* did not affect the siRNA levels but decrease the Pol V transcripts, which suggested that RDM16 functions in a later step of RdDM through a different mechanism from that of SR45. In addition, we performed genome-wide DNA methylation analysis and found that the majority of loci whose methylation is RDM16-dependent overlapped with Pol IV- and ROS1-targeted loci. Together, our results shed new light on the RdDM pathway and the dynamic balance between RdDM and active DNA demethylation.

## Results

### Identification of the *rdm16* mutant through the screening of *ros1* suppressors

**Figure 1:**
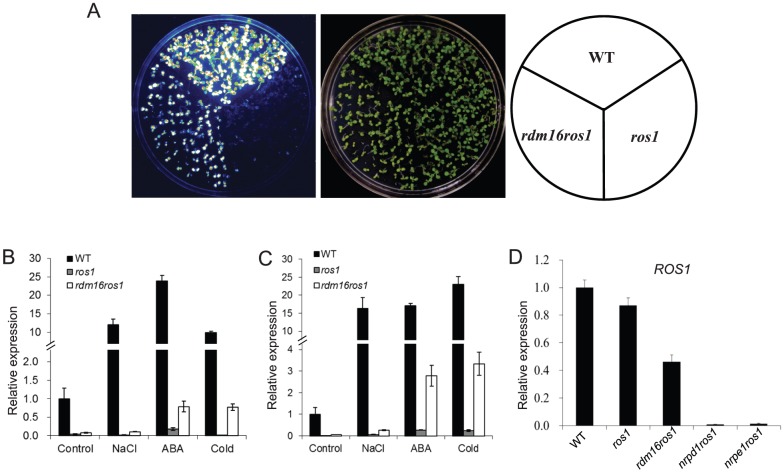
Mutation of *RDM16* partially releases the transcriptional silencing of *RD29A-LUC* transgene and endogenous *RD29A* in *ros1* mutant background. (**A**) Luminescence imaging of *RD29A-LUC* expression in WT, *ros1* and *rdm16ros1*. (**B–C**) Real-time RT-PCR analysis of the expression of *RD29A-LUC* (**B**) and endogenous *RD29A* (**C**) in WT, *ros1* and *rdm16ros1* exposed to NaCl, abscisic acid (ABA) and cold (4°C) stresses. (**D**) Expression analysis of *ROS1* in WT, *ros1*, *rdm16ros1*, *nrpd1ros1* and *nrpe1ros1*.

To identify components involved in the RdDM machinery, we carried out a forward genetics screen on a T-DNA-mutagenized population in the *ros1* background, which contains the *RD29A* promoter-driven luciferase reporter gene (*RD29A-LUC*) as well as the *35S* promoter-driven *NPTII* transgene (*35S-NPTII*) [41]. In this system, the *RD29A-LUC* transgene, endogenous *RD29A* and *35S-NPTII* transgene are well expressed in wild-type plants under stress conditions, while mutations in *ROS1*, the 5-methylcytosine DNA glycosylase/DNA demethylase gene, led to the silencing of all the three genes. Based on reactivation of the *RD29A-LUC* in *ros1*, we have identified a number of genes required for RdDM [28], [29], [42]–[44]. In this study, we identified a new mutant, *rdm16*, as a suppressor of *ros1* (Figure 1A). Mutation of *RDM16* in the *ros1* background caused a partial release of the silencing of *RD29A-LUC* under stress treatment. Nevertheless, the silencing of the RdDM-independent *35S-NPTII* transgene was not released in *rdm16ros1* compared to the *ros1* single mutant (Figure S1A).

We also performed quantitative RT-PCR for the expression analysis of *RD29A-LUC* and endogenous *RD29A* under various stress conditions. The results showed that mutation of *RDM16* reactivated the expression of both *RD29A-LUC* and endogenous *RD29A* under salt, ABA and cold treatments (Figures 1B and 1C).

### The *rdm16* mutation causes reduced DNA methylation and morphological and physiological defects

**Figure 2:**
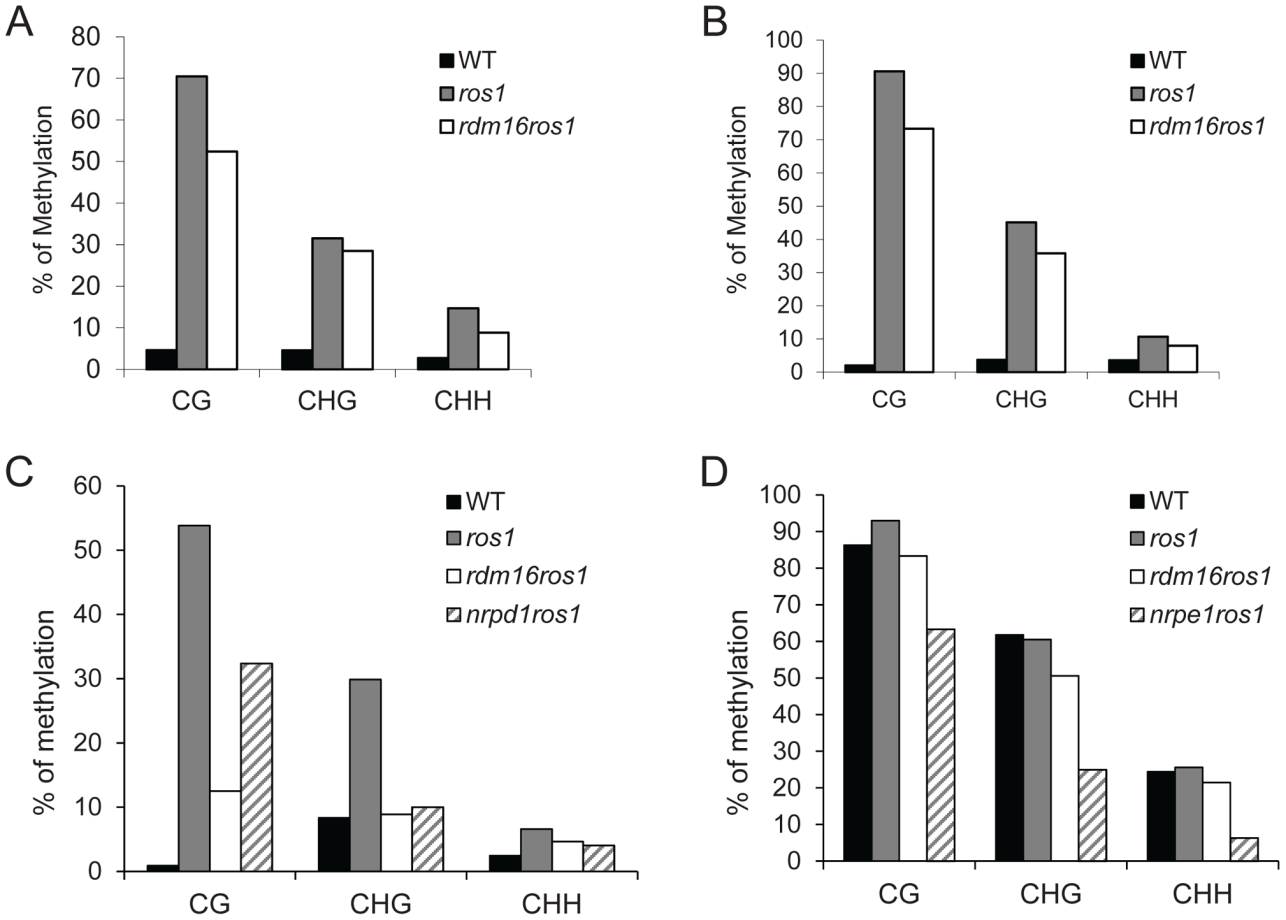
Cytosine DNA methylation analysis of transgenic and endogenous loci in WT, *ros1*, *rdm16ros1*, *nrpd1ros1* and *nrpe1ros1* through bisulfite sequencing. DNA methylation analysis of transgenic *RD29A* promoter (**A**), endogenous *RD29A* promoter (**B**), At4g18650 promoter (**C**) and AtSN1 (**D**).

To examine the cause for the reactivation of *RD29A-LUC* expression in the *rdm16ros1* mutant, we carried out DNA methylation analysis by bisulfite sequencing of WT, *ros1* and *rdm16ros1* mutants. Consistent with previous reports, the DNA methylation of both *RD29A-LUC* and endogenous *RD29A* promoters was detected at low levels in WT, while heavy cytosine methylation at all sequence contexts (CG, CHG and CHH) was found in *ros1* (Figures 2A and 2B). In comparison with *ros1*, the high methylation of both *RD29A-LUC* and endogenous *RD29A* promoters was substantially decreased in the *rdm16ros1* double mutant at all the three cytosine contexts CG, CHG and CHH. We also measured the DNA methylation in the *35S* promoter and the results showed that the DNA methylation level of *35S* promoter in *rdm16ros1* was similar to that in *ros1* (Figure S1B), which is consistent with the phenotype that mutation of *RDM16* did not affect the silencing of *35S-NPTII* transgene in *ros1* (Figure S1A). Additionally, we also performed DNA methylation analysis on a ROS1-targeted endogenous locus, At4g18650 [45], and RdDM-targeted repeat loci, *AtSN1*, *SoloLTR* and *MEA-ISR*. Results showed that DNA methylation of At4g18650 promoter and *AtSN1* was reduced in *rdm16ros1* at all three cytosine contexts (CG, CHG and CHH) in comparison with that in *ros1* (Figures 2C and 2D). The decreased level of DNA methylation in At4g18650 promoter was comparable to or even stronger in *rdm16ros1* than that in *nrpd1ros1* (Figure 2C). Mutation of *RDM16* also reduced the DNA methylation at CHG sites of *SoloLTR*, while its effect on CG and CHH was not observed (Figure S2A). *rdm16ros1* did not affect the DNA methylation at *MEA-ISR* locus (Figure S2B). Previous reports revealed that the expression of *ROS1* is sensitive to RdDM mutations [29], [42]–[44]. Our expression analysis showed that like previously reported RdDM mutants, the expression of *ROS1* was also reduced in *rdm16ros1* (Figure 1D), which is consistent with a role of *RDM16* in the RdDM pathway.

**Figure 3:**
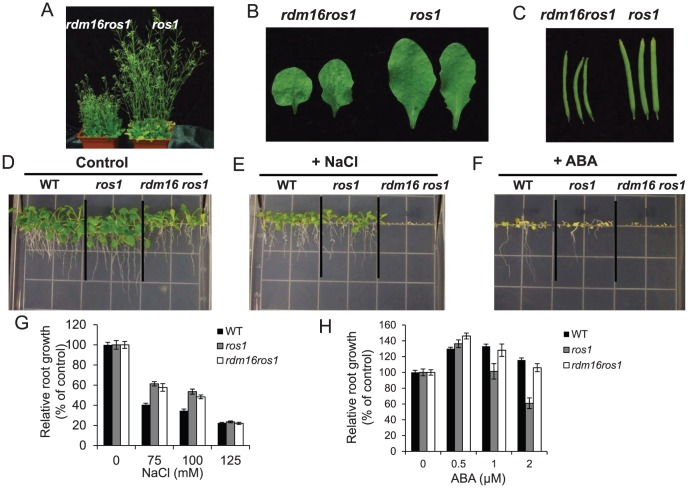
Morphological and physiological defects in *rdm16ros1* compared to *ros1* mutant. (**A–C**) Morphological comparison of *rdm16ros1* with *ros1* with respect to plant stature (**A**), leaves (**B**) and siliques (**C**). (**D–F**) WT and *ros1*, *rdm16ros1* seed germination under control condition (**D**), 75 mM NaCl (**E**) and 0.5 µM ABA (**F**). (**G–H**) Seeds were germinated on a half-strength MS plate for 6 d and then the seedlings were exposed to different concentrations of NaCl (**G**) and ABA (**H**) for 7 d. Data are means ± SD (n = 15).

Mutation of *RDM16* also caused morphological defects, including dwarf stature, smaller, rounded and wrinkled leaves, and smaller siliques (Figures 3A–3C). Furthermore, seed germination of *rdm16ros1* mutant was hypersensitive to salt stress and abscisic acid (ABA) (Figures 3D–3F). Nevertheless, at the young seedling stage, *rdm16ros1* did not show higher sensitivity to salt and ABA than *ros1* and WT (Figures 3G and 3H).

### Cloning of *RDM16*

To perform genetic analysis for *rdm16ros1*, we generated an F2 population from a cross between *rdm16ros1* and *ros1* and then observed the LUC signal and morphological defects. We found that reactivation of LUC expression was tightly linked to the morphological defects. Among 235 F2 plants examined, 59 plants showed both increased LUC signal and morphological defects, while the rest 176 plants showed low LUC signal and normal morphology. The ratio of plants with high LUC expression to plants with low LUC expression fitted to 1∶3 (P>0.95), suggesting that the increased LUC expression and morphological defects was controlled by a recessive mutation in a single nuclear gene.

To map the responsible gene, we used an F2 population derived from a cross between *rdm16ros1* (C24 background) and *ros1-4* (Col background) and selected plants with defective morphology and increased LUC signal for mapping. By using 25 indel markers across the genome and 74 plants, we were able to find the marker At124 at 11.3 Mb of chromosome 1 was linked to the *RDM16* gene (Figure S3A). By developing three more polymorphic markers around At124, we mapped the gene between At120 and At124 on chromosome 1 with a recombination rate of 8.7% and 4.7% from the *RDM16* gene, respectively. To further map the gene, we used a population of 632 plants and developed additional 6 polymorphic markers between the At120 and At124 markers. Through linkage analysis, we further mapped the *RDM16* gene between the At126 and At130 markers, with 13 and 1 recombinants, respectively (Figure S3B). Three markers within the two markers At126 and At130 were tightly linked to the gene. As a result, the candidate region for *RDM16* was defined to about 1.39 Mb (Figure S3B).

To clone the gene, we sequenced the whole genome of *rdm16ros1* mutant by second-generation high throughput DNA sequencing. In the 1.39 Mb mapping interval, we found a gap in the promoter of At1g28060 in *rdm16ros1* mutant, suggesting a deletion or insertion occurred in the region (Figure S4A). PCR analysis showed that the DNA fragment spanning the gap could not be amplified in *rdm16ros1*, whereas it was well amplified in WT and *ros1* mutant (Figure S4B). These results suggested that there was a DNA fragment inserted into the At1g28060 promoter of *rdm16ros1* mutant. To determine the DNA sequence inserted into the promoter, we performed TAIL-PCR analysis on both sides of the insertion. We detected partial sequence of At1g24590 from this analysis, although the full sequence of the insertion was not known (Figure S4C). In addition, we found that the insertion also caused a 45-bp deletion (-154 to -109 bp from ATG) in the promoter of At1g28060. To examine the effect of the mutation on the expression of At1g28060, we compared mRNA expression level of the gene between *rdm16ros1* and *ros1*. In comparison with *ros1*, mRNA expression of the coding sequence of the gene was decreased by about 2 fold in *rdm16ros1*, and the expression of 5′UTR was greatly reduced (Figure S5). These results suggest that decreased expression of At1g28060 might be responsible for the observed phenotypes in *rdm16ros1*.

To confirm that At1g28060 is *RDM16*, two T-DNA insertion lines (*rdm16-2* and *rdm16-3*) were obtained and characterized. *rdm16-2* has a T-DNA insertion in the fifth intron of At1g28060 (Figures S6A and S6B). The T-DNA insertion did not abolish the expression of the full mRNA of the gene, but reduced its expression (Figures S6C and S6D). *rdm16-2* also showed similar defective leaf phenotype to *rdm16-1*, including smaller, rounded and wrinkled leaves, although the morphological defects in *rdm16-2* were stronger than those in *rdm16-1* (Figure S6E). To examine whether *rdm16-2* affected DNA methylation, we compared the DNA methylation level of *AtSN1* between WT and *rdm16-2* through bisulfite sequencing. Like *rdm16-1*, *rdm16-2* also decreased DNA methylation at all three cytosine contexts (CG, CHG and CHH) in comparison with its WT (Figure S6F). Furthermore, the expression of *ROS1* was reduced in *rdm16-2* as well (Figure S6G).

The *rdm16-3* mutant contains a T-DNA insertion in the fifth exon of At1g28060 (Figure S6A. Genomic PCR analysis with selfed progeny of a heterozygous plant of *rdm16-3* showed that no homozygous *rdm16-3* plants were found among 107 selfed progeny, suggesting that homozygous *rdm16-3* mutant was lethal. In addition, we found wild-type and heterozygous plants at a 68∶39 ratio, distorting from the expected ratio of 1∶2, which suggested that the viability of male and/or female gametes of the heterozygous plants was affected. To test this hypothesis, we made reciprocal crosses between heterozygous *rdm16-3*/+ and *SALK_057447C* insertion line, and then analyzed the frequency of *rdm16-3* haploid in the resultant F1 plants. The *SALK_057447C* line did not show morphological defects and its T-DNA insertion site could be used to determine whether the F1 plants are from real crosses. Of 107 real F1 plants with *SALK_057447C* line as the pollen donor, 17 plants were heterozygous for *rdm16-3*, whereas 90 plants showed wild-type *RDM16*. Among 82 F1 plants with *rdm16-3*/+ as the pollen donor, there were 24 heterozygous and 58 wild-type plants. These results indicated that knockout of *RDM16* in both female and male gametes reduced their viability and the effect on female gametes was more severe.

**Figure 4:**
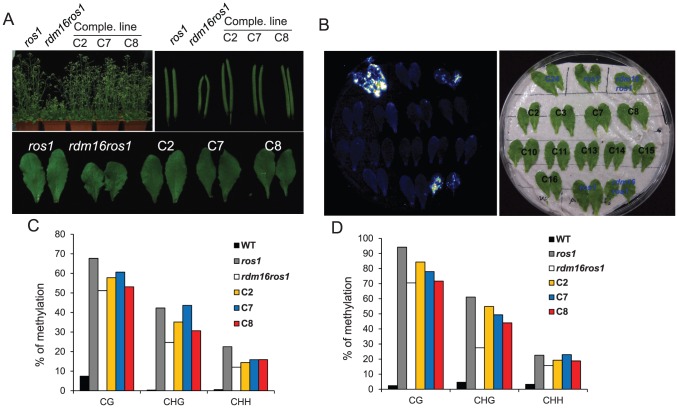
Complementation test for the *rdm16ros1* mutant. (**A**) All the morphological defects including dwarf stature, smaller silique and leaves in *rdm16ros1* were rescued in three independent complementation lines. (**B**) Luminescence imaging of detached leaves exposed to 2% NaCl for 3 h. The increased expression of *RD29A-LUC* in *rdm16ros1* was re-silenced in all complementation lines. (**C–D**) Cytosine DNA methylation analysis of transgenic *RD29A* promoter (**C**) and endogenous *RD29A* promoter (**D**) in WT, *ros1*, *rdm16ros1* and complementation lines by bisulfite sequencing.

To further confirm that the mutation in At1g28060 is responsible for the observed phenotype in *rdm16ros1*, we conducted a complementation test on *rdm16ros1* by introducing a wild-type *RDM16* with 1.4 kb promoter and the full genomic sequence of the gene into the mutant. In the complementation lines, the morphological defects including dwarf stature, smaller and deformed siliques, and defective leaves were fully complemented in the T2 generation (Figure 4A). We also analyzed the *RD29A-LUC* expression by luminescence imaging and DNA methylation level of both *RD29A-LUC* and endogenous *RD29A* promoters by bisulfite sequencing in the complementation lines. Results showed that the increased expression of *RD29A-LUC* and the reduced DNA methylation level of both promoters in *rdm16ros1* were rescued in the complementation lines (Figures 4B–4D). Taken together, these results show that At1g28060 is *RDM16* and the mutation in At1g28060 is responsible for the altered expression and DNA methylation of reporter gene and endogenous loci, and the defective plant development in *rdm16ros1*.

### *RDM16* encodes a pre-mRNA splicing factor

*RDM16* has six exons and five introns, encoding a peptide of 786 amino acids with a predicted molecular mass of 88.6 kD. BLAST searches indicated that *RDM16* encodes a pre-mRNA-splicing factor 3, a component of U4/U6 snRNP protein complex. Domain analysis predicted that RDM16 has a pre-mRNA processing factor 3 (PRP3) domain and a DUF1115 domain with unknown function (Figure S7). RDM16 is conserved in eukaryotes and has putative orthologs in rice, human and yeast (Figure S7). For example, RDM16 exhibits 26.8% identity and 46.0% similarity at the amino acid level to human HPRP3 (accession number NP_004689), whose mutation results in autosomal dominant retinitis pigmentosa in humans [46]. RDM16 has 30.2% similarity to yeast Prp3 (accession number NP_010761), which is a U4/U6 snRNP protein necessary for the integrity of U4/U6 snRNP and U4/U6.U5 tri-snRNP [47]. In addition, there is a homologous protein, At3g55930, sharing 40.2% identity with RDM16, encoded in the *Arabidopsis* genome.

To examine whether RDM16 is indeed involved in pre-mRNA splicing, we carried out Illumina paired-end RNA-seq in WT (Col) and *rdm16-2* mutant seedlings. A total of 51.5 million and 53.2 million reads for WT and *rdm16-2* with the average length of 90 nucleotides were generated, respectively. Among them, there were 48.3 million and 49.6 million unique reads for WT and *rdm16-2*, respectively, mapped to the *Arabidopsis* genome. Analysis of intron-retention events in WT and *rdm16-2* showed that 308 intron-retention events in 258 genes occurred in *rdm16-2* mutant in comparison with WT (FDR<0.01) (Table S1). This result indicated that RDM16 is involved in pre-mRNA splicing *in planta*.

Our RNA-seq analysis also showed that there were 689 and 152 genes significantly upregulated (>2 fold) and downregulated (<2 fold) in the *rdm16-2* mutant, respectively (Tables S2 and S3). Although the expression of so many genes was altered, no genes involved in the RdDM pathway were found in the list. Additionally, we did not find any genes involved in the RdDM pathway showing splicing defects in the mutant (Table S1). Therefore, it is unlikely that the altered DNA methylation in the *rdm16* mutants was an indirect consequence of reduced expression or splicing defect of genes encoding RdDM components. To further exclude the possibility of RDM16 affecting the expression or splicing of the RdDM components, we carried out real-time RT-PCR and regular RT-PCR to examine the expression of the RdDM components among WT, *ros1* and *rdm16ros1* by using primers spanning introns of the genes. The real-time RT-PCR analysis showed that the expression of the genes involved in the RdDM pathway was not different in the three lines (Figure S8A). No splicing defects in any RdDM pathway genes were found in the *rdm16ros1* mutant by the RT-PCR analysis (Figure S8B). Together, these results indicated that the involvement of RDM16 in RdDM was not an indirect effect of RDM16 on the expression or splicing of RdDM components.

### Genome-wide effects of RDM16 on DNA methylation

**Figure 5:**
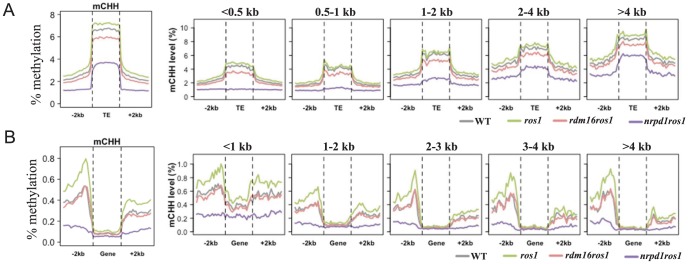
Average CHH methylation levels in transposable elements (TEs) (A) and genes (B). TEs and genes were divided into 5 groups based on their size for detailed comparison of the DNA methylation levels among WT, *ros1*, *rdm16ros1* and *nrpd1ros1*.

To examine the effect of RDM16 on the *Arabidopsis* methylome, we performed whole genome bisulfite sequencing in WT, *ros1*, *rdm16ros1* and *nrpd1ros1* (4-week-old leaves). The average depth of sequenced methylomes is 15–18 with 0.3–0.5% error rates, which indicated a high quality of the data (Table S4). We analyzed the methylation levels of transposable elements (TEs), genes, and 2 kb upstream or downstream of the genes or TEs. Transposable elements are heavily methylated and show higher methylation levels than surrounding regions at all sequence contexts (Figure 5A, Figure S9A). In *ros1* mutant, the overall CG and CHH methylation of TEs and surrounding regions were slightly increased in comparison with the wild-type. Mutation of *RDM16* did not affect the CG and CHG methylation (Figure S9A). However, CHH methylation of both TEs and surrounding regions was substantially decreased in *rdm16ros1* in comparison with *ros1* or WT (Figure 5A).

To investigate whether RDM16 has preference for TEs of different sizes, we divided the TEs into five groups based on the TE size and calculated average DNA methylation levels. The DNA methylation level of TEs was gradually elevated as the TE size increased. Mutation of *RDM16* reduced the CHH methylation of all five groups of TEs, but did not influence CG and CHG methylation (Figure 5A, Figure S9A). This result indicates that RDM16 targets all TEs and does not have a preference for TEs of different sizes. The methylation pattern of TEs in *rdm16ros1* is similar to *nrpd1ros1*, although the decrease in the level of DNA methylation was less dramatic in *rdm16ros1* (Figure 5A).

In contrast to TEs, gene body methylation was mainly in the CG sequence context and the CG methylation is depleted towards both the 5′ and 3′ ends of genes (Figure S9B), which suggested that CG methylation at the 5′ and 3′ ends of the genes might not be compatible with Pol II-mediated transcription. Mutation of *RDM16* did not affect overall DNA methylation of gene bodies in any sequence context (Figure 5B, Figure S9B). The methylation level of gene body was also not reduced in the *nrpd1ros1* mutant, suggesting that the gene body methylation is largely independent of RdDM. Nevertheless, when we divided the genes into 5 groups with different gene sizes for the methylation analysis, we found that CHH methylation of short genes (<1 kb) was reduced in *rdm16ros1* as well as *nrpd1ros1* compared to *ros1* mutant (Figure 5B, Figure S9B). Short genes have low CG methylation and increased CHH methylation, which suggested that RdDM pathway preferentially targets short genes, which results in elevated CHH methylation, and RDM16 is involved in the regulation of the DNA methylation of short genes. The CHG and CHH methylation levels of gene surrounding regions were greater than that of gene body, and 5′ promoter regions had higher methylation level than 3′ downstream region of genes in the wild-type (Figure 5B, Figure S9B). In *ros1* mutant, the DNA methylation level was increased in all sequence contexts and the effect on CHH methylation was greater, suggesting that ROS1 targeted the promoters and downstream sequences of genes. Additionally, as gene size increased, the difference in CHH methylation in 3′ downstream regions of genes was decreased between WT and *ros1* mutant (Figure 5B), which suggested that ROS1 preferentially targeted 3′ downstream regions of short genes. Mutation of *RDM16* did not affect the CG methylation, but slightly reduced the CHG methylation and notably decreased the CHH methylation in both 5′ and 3′ regions of genes (Figure 5B, Figure S9B). In the *rdm16ros1* mutant, the CHH methylation of 5′ and 3′ regions of genes of all sizes was reduced to a similar level as in WT, while in the *nrpd1ros1* mutant the CHH methylation was further decreased (Figure 5B). These results suggest that ROS1 actively counteracts the effect of RDM16 and the RdDM pathway on the DNA methylation of 5′ promoters and 3′ downstream regions of genes.

**Figure 6:**
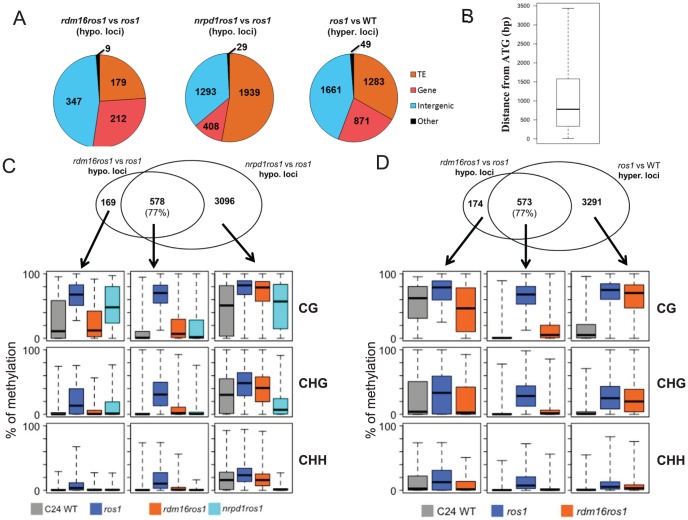
Analysis of DMRs identified in *rdm16ros1*. (**A**) Category of hypomethylated or hypermethylated loci in *rdm16ros1*, *nrpd1ros1* or WT in comparison with *ros1*. (**B**) Distance distribution of intergenic DMRs relative to gene start codon. (**C–D**) Overlap of differentially methylated loci between *rdm16ros1* and *nrpd1ros1* (**C**), and between *rdm16ros1* and *ros1* (**D**). Boxplots represent methylation levels of each class of differentially methylated loci.

We next identified differentially methylated regions (DMRs) in *rdm16ros1* compared to *ros1* mutant. There were 747 loci with decreased DNA methylation and 468 loci with increased DNA methylation in *rdm16ros1* in comparison with *ros1* mutant (Tables S5 and S6), while the number of hypomethylated and hypermethylated loci in *nrpd1ros1* was 3929 and 1417, respectively (Tables S7 and S8). Of the 747 hypomethylated loci in *rdm16ros1*, 347 loci were located in intergenic regions, and most of the 347 loci were located within 1.5 kb promoter regions (Figures 6A and 6B), which suggested that RDM16 was preferentially involved in the regulation of DNA methylation of intergenic regions and potentially involved in regulating gene transcription. In contrast to RDM16, transposable elements (TE) are the main targets of NRPD1 (Figure 6A). Despite their different preferences, the hypomethylated loci of *rdm16ros1* largely overlapped (77%) with those of *nrpd1ros1* (Figure 6C). Moreover, the non-overlapping hypomethylated loci of *rdm16ros1* also showed reduced DNA methylation levels in *nrpd1ros1*, especially at the CHH context. This result suggests that all RDM16 targets are influenced by the dysfunction of NRPD1, although some RDM16 targets were excluded from the list of NRPD1 targets due to the stringent cutoffs used to define differentially methylated regions. Interestingly, we noticed that the DNA demethylation enzyme ROS1 also preferentially targeted intergenic regions (Figure 6A). When comparing the 747 hypomethylated loci in *rdm16ros1* with the hypermethylated loci in *ros1*, we found a striking overlap between them (Figure 6D). Similar to the case in *nrpd1ros1*, the nonoverlapping hypomethylated loci of *rdm16ros1* also showed altered DNA methylation in *ros1*. Together, these results suggest that RDM16 is involved in the RdDM pathway and preferentially regulates the DNA methylation of loci that are targeted for demethylation by ROS1.

**Figure 7:**
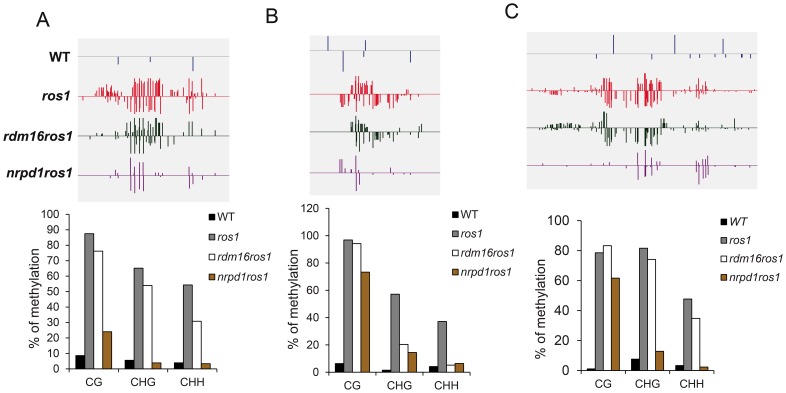
DNA methylation analysis of selected loci in WT, *ros1*, *rdm16ros1* and *nrpd1ros1*. (**A–C**) DNA methylation levels of three loci. Upper panel, DNA methylation snapshot in IGV browser from whole-genome bisulfite sequencing data; Lower panel, individually bisulfite sequencing results. (**A**) At5g42940 promoter, (**B**) At5g35730 promoter, (**C**) At1g26400 promoter.

To validate the whole-genome DNA methylation results, we selected 3 from the 747 hypomethylated loci for individual locus bisulfite sequencing. All three loci showed low levels of DNA methylation in WT, but became heavily methylated in *ros1* at all sequence contexts (Figure 7). In the *rdm16ros1* double mutant, the high DNA methylation was reduced compared to the *ros1* single mutant, especially at the CHG and CHH contexts, although the decreased level of DNA methylation in *rdm16ros1* was less prominent than in *nrpd1ros1* (Figure 7).

### The *rdm16* mutation does not affect siRNA abundance

**Figure 8:**
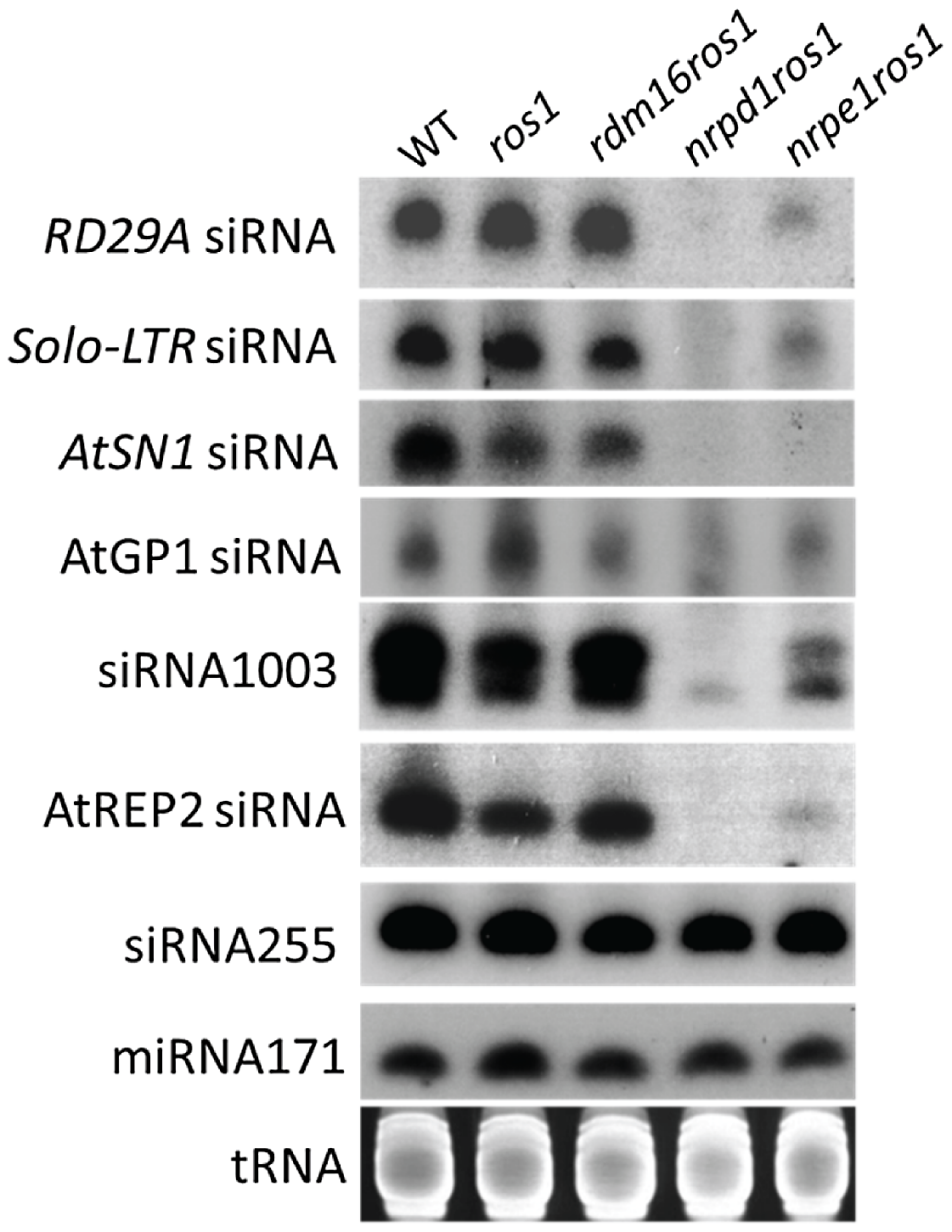
siRNA accumulation was not affected by the mutation of *RDM16*. Small RNA Northern blotting of various siRNAs in WT, *ros1*, *rdm16ros1*, *nrpd1ros1* and *nrpe1ros1*. tRNA, miRNA171 and siRNA255 were used as loading controls.

To investigate whether *rdm16* may alter DNA methylation through influencing small RNA levels, we performed small RNA Northern blot analysis in WT, *ros1*, *rdm16ros1*, *nrpd1ros1* and *nrpe1ros1*. The results showed that the *rdm16* mutation did not affect the accumulation of any of the tested siRNAs compared to WT and *ros1* controls, although the abundance of these tested siRNAs was greatly reduced in both *nrpd1ros1* and *nrpelros1* mutants (Figure 8). The targets of these siRNAs include the *RD29A* promoter, Solo-LTR and AtSN1 whose methylation levels were decreased in the *rdm16ros1* mutant (Figure 2, Figure S2A). Therefore, these results suggest that RDM16 regulates DNA methylation not through influencing the siRNA abundance.

### RDM16 regulates Pol V transcript levels

**Figure 9:**
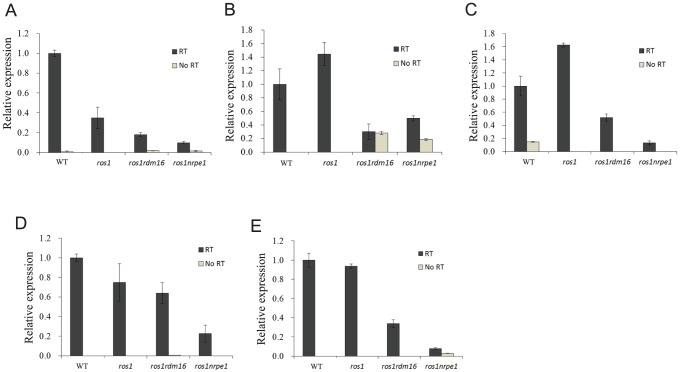
The *rdm16* mutation reduced the levels of Pol V transcripts. (**A–E**) Five Pol V-dependent loci showed decreased Pol V transcript levels in *rdm16ros1* compared to WT and *ros1* controls. (**A**) RD29A promoter, (**B**) IGN5, (**C**) IGN28, (**D**) IGN30, (**E**) IGN32.

The lack of effect of the *rdm16* mutation on siRNA abundance suggests that RDM16 might function at a later step in the RdDM pathway. Therefore, we examined whether the *rdm16* mutation may affect DNA methylation by influencing Pol V transcript levels. We selected 13 Pol V-dependent loci [48] including the *RD29A-LUC* promoter for analysis. Pol V-dependent transcripts of 8 of the 13 loci were detected in the C24 wild type under our conditions. Five of the 8 loci including the *RD29A-LUC* promoter displayed a decreased expression in *rdm16ros1* compared to the WT and *ros1* controls, although the reduction in expression level was less in *rdm16ros1* than in *nrpe1ros1* (Figure 9). All of the five loci with decreased Pol V transcripts in *rdm16ros1* showed a reduced DNA methylation level to some extent in *rdm16ros1* compared to *ros1* (Figures 2A and 2B, Figure S10). We also compared the levels of the Pol V-dependent transcripts in Col WT, *rdm16-2* and *nrpe1-11*. The results show that 8 loci had a decreased expression in *rdm16-2* compared to WT, although the reduction in *rdm16-2* was not as dramatic as in *nrpe1-11* (Figure S11). These data suggest that RDM16 regulates DNA methylation through influencing Pol V transcripts.

**Figure 10:**
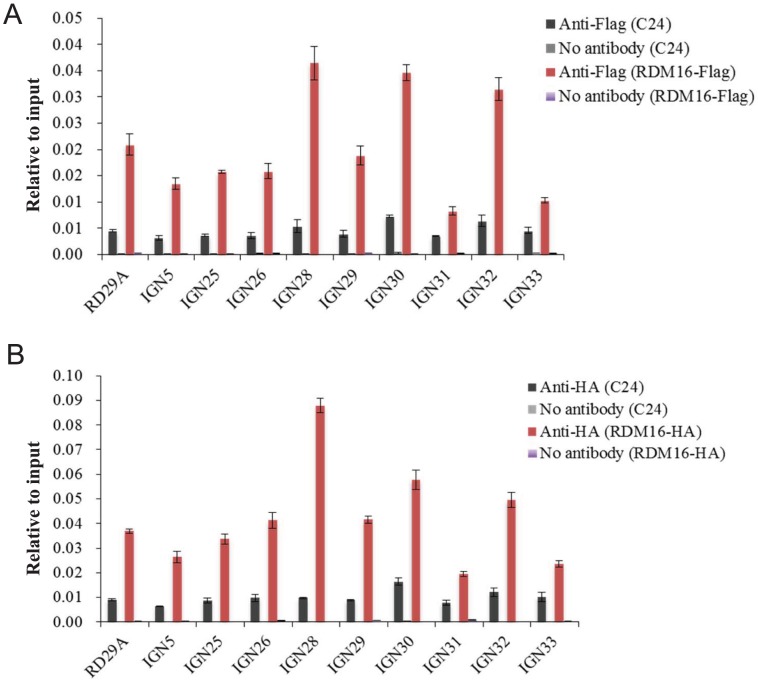
ChIP analysis of RDM16 on Pol V targeted loci. (**A**) ChIP analysis of RDM16-3xFlag transgenic lines using anti-Flag antibody. (**B**) ChIP analysis of RDM16-3xHA transgenic lines using anti-HA antibody.

To investigate whether RDM16 may be associated with its target loci, we performed chromatin immunoprecipitation (ChIP) assays on Pol V target loci by using native promoter-driven RDM16-3xFlag or RDM16-3xHA transgenic lines. The RDM16-3xFlag or RDM16-3xHA construct complemented the defects of *rdm16* mutant and we were able to detect tagged RDM16 protein in the transgenic lines by Western blot analysis (Figure S12), indicating that the tagged proteins are expressed and functional *in vivo*. The ChIP results show that RDM16 was enriched at all of the target loci tested in both RDM16-3xFlag and RDM16-3xHA transgenic lines (Figure 10), which indicated that RDM16 is associated with its target loci.

To examine the subnuclear localization patterns of RDM16, we used the RDM16-3xFlag transgenic line to perform immunolocalization assays. The results show that the RDM16 protein was dispersed throughout the nucleoplasm without any preferential accumulation at the Cajal body (Figure S13), based on the co-localization analysis between RDM16 and U2B, a maker for the Cajal body [26]. This localization pattern of RDM16 differs from that of ZOP1, a splicing factor recently reported to be involved in the RdDM pathway through affecting Pol IV-dependent siRNA accumulation [49]. In addition to the dispersed nucloplasmic localization, ZOP1 also preferentially accumulates in the Cajal body. The presence of ZOP1 but not RDM16 in the Cajal body is consistent with our hypothesis that RDM16 regulates DNA methylation through a different mechanism from those of reported splicing factors.”

## Discussion

In this study, we isolated a new factor, RDM16, that functions in the RdDM pathway through a forward genetic screen. RDM16 is required for the transcriptional silencing and the increased DNA methylation of transgenic *RD29A-LUC* in *ros1* mutant. Our genome-wide DNA methylation analysis showed that RDM16 also affects DNA methylation of TEs and gene surrounding regions globally and RDM16 preferentially influences NRPD1- and ROS1-targted loci. RDM16 encodes a homolog of yeast Prp3 protein, which is a component of U4/U6 snRNP-associated protein and is involved in pre-mRNA-splicing in yeast [47]. Our RNA-seq data indicated that RDM16 is involved in the pre-mRNA-splicing in *Arabidopsis* (Table S1). None of the mis-spliced genes had changes in DNA methylation in the *rdm16* mutant, suggesting that splicing and regulation of DNA methylation are two separate functions of RDM16. However, there is a possibility that RDM16 regulates DNA methylation by influencing expression and/or pre-mRNA splicing of genes involved in the RdDM pathway. Our RNA-seq and RT-PCR analysis indicated that RDM16 did not affect the expression or pre-mRNA splicing of genes encoding known RdDM components (Tables S1, S2, S3, Figure S7). These suggest that RDM16 involvement in the regulation of DNA methylation might be quite direct. To examine the mechanism of RDM16 in the RdDM, we compared the expression level of small RNAs and Pol V transcripts. However, unlike previously reported putative splicing factor *sr45*, mutation of *RDM16* did not influence the small RNA accumulation but reduced the expression level of Pol V transcripts (Figures 8 and 9, Figure S11), which suggested that RDM16 regulates DNA methylation through a different mechanism from SR45.

RDM16 is critical for normal plant development, which is likely due to its role in the splicing of developmentally important genes. Knockout of *RDM16* in both female and male gametes reduced their viability and knockout of both maternal and paternal *RDM16* alleles in the embryo is lethal. Knockdown of *RDM16* caused a series of developmental defects, including reduced plant stature, smaller leaves and siliques, rounded and wrinkled leaves (Figures 3A–C, Figure S6E). Furthermore, Knockdown of *RDM16* led to an increased sensitivity to salt stress and ABA in seed germination (Figures 3D–F). Dysfunction of *STA1*, the U5 snRNP-associated pre-mRNA-splicing factor, also caused an increased sensitivity to ABA [50]. These suggest that hypersensitivity to ABA might be a common feature in the splicing factor-defective mutants. Nevertheless, there are differences in the physiological defects between the *rdm16* and *sta1* mutants. The hypersensitivity to chilling in *sta1* mutant was not observed in the *rdm16* mutant. On the other hand, *sta1* did not have the phenotype of hypersensitivity to salt stress as *rdm16* did. Moreover, unlike in *sta1*, the hypersensitivity to salt stress and ABA was not observed in the *rdm16* mutant at the seedling stage (Figures 3G and 3H). Since both *rdm16-1* and *sta1-1* are weak mutant alleles but are not nulls, it is not clear whether the different physiological phenotypes between *rdm16-1* and *sta1-1* were due to the different mutations or due to the distinct targets of RDM16 and STA1.

In fission yeast, the formation of heterochromatin requires the RNAi machinery whose core components consist of Dicer (Dcr1), Argonaute (Ago1) and RNA-dependent RNA polymerase (Rdp1) [51], [52]. The pathway of RNA-induced heterochromatin formation in fission yeast parallels the RdDM pathway in *Arabidopsis*. In the fission yeast, Pol II transcribes the *dg* and *dh* repeats of centromeric regions and then Rdp1-containing RNA-dependent RNA polymerase complex (RDRC) and Dcr1 process the centromeric transcripts into siRNAs [51], [52]. The siRNA is loaded into Ago1 to form the RNA-induced transcriptional gene-silencing (RITS) complex that also contains the chromodomain protein Chp1 and GW-motif-containing protein Tas3. Through base-pairing of the siRNA with Pol II-produced nascent repeat transcripts, the RITS is specifically recruited to the target region and facilitate the recruitment of histone-modifying enzymes such as the H3K9 methyltransferase Clr4 to induce H3K9 methylation and the formation of heterochromatin [51], [52]. Recently, several splicing factors were reported to be involved in the RNAi-directed silencing process in fission yeast [53], [54]. Mutation of these splicing factors, but not the splicing itself, affects the accumulation of centromeric siRNAs and consequently the integrity of centromeric heterochromatin. Splicing factors are found to be co-purified with affinity-selected Cid12, a component of RDRC, and therefore it was proposed that splicing factors might be recruited to centromeric noncoding RNA through the recognition of *dg* intron sequence and then interact with RDRC to provide a platform for siRNA generation and finally facilitate the centromere repeat silencing [53], [54]. Among these splicing factors involved in the centromere repeat silencing, Prp3, the homolog of RDM16 in fission yeast, is not included. Nevertheless, Prp3 was co-immunoprecipitated with FLAG-tagged Cid12 and mutation of *Prp3* led to the increased accumulation of *dg* transcripts [53], [54], which suggest that Prp3 is also involved in the processing of the centromeric transcripts into siRNAs, but the influence may not be strong enough to have an observable effect on the centromere repeat silencing in the mutant. In this report, we showed that RDM16, a homolog of yeast Prp3, is involved in the RdDM pathway in *Arabidopsis*. Mutation of *RDM16* reduced the DNA methylation and released the transcriptional silencing of transgene and endogenous targets. However, *rdm16* mutation did not influence the siRNA accumulation at DNA methylation-affected loci. These results suggest that RDM16 function in the RdDM pathway might adopt a different mechanism from the yeast splicing factors in the pathway of centromere repeat silencing.

Mutation of *SR45* causes a decreased accumulation of siRNAs and the AGO4 protein level was also reduced, which suggested that SR45 acts at an early step in the RdDM pathway in siRNA generation [36]. Recently, Zhang et al. (2013) reported that ZOP1 and several other splicing factors are also involved in regulating DNA methylation through influencing siRNA abundance [49]. Since the *rdm16* mutation did not cause a reduction in siRNA abundance, this suggests that RDM16 likely functions in a later step in RdDM. Indeed, mutation of *RDM16* caused a reduction in the levels of Pol V transcripts. Dysfunction of Pol V not only abolishes the Pol V transcripts but also influences the siRNA abundance. However, the *rdm16* mutation caused a decrease in Pol V transcript levels but did not affect the siRNA abundance, suggesting that the reduction in the levels of Pol V transcripts in *rdm16* may not be strong enough to cause a decrease in the siRNA accumulation or that Pol V may function in the siRNA accumulation independently of its transcripts. As a splicing factor, RDM16 has a known role in RNA processing, so it is possible that RDM16 function in RdDM may involve an interaction with scaffold RNAs generated by Pol V or Pol II. Consistent with this hypothesis, our ChIP assays indicated that RDM16 is associated with Pol V target loci. The association of RDM16 with Pol V target loci might be mediated through the interaction of RDM16-containing complex with Pol V or nascent Pol V transcripts. Further work is required to determine whether RDM16 is associated with Pol V and/or can interact with Pol V transcripts. Regardless, the fact that SR45 [36] and ZOP1 [49] but not RDM16 are involved in siRNA accumulation suggests that the various splicing factors function at different steps in the RdDM pathway. This is consistent with our notion that RDM16 functions directly in RdDM, and further argues against the model that splicing factors function indirectly in RdDM by affecting the pre-mRNA splicing of genes encoding RdDM components.

Our analysis of the methylome of the *rdm16* mutant showed that dysfunction of *RDM16* affected the overall CHH methylation of TEs (Figure 5A), indicating an important role of RDM16 on the silencing of parasitic sequences. The overall methylation of gene body was not influenced by the mutation of any of the genes, *ROS1*, *RDM16* or *NRPD1* (Figure 5B). However, in short genes (<1 kb), mutation of all three genes affected the genic CHH methylation levels, and the methylation level was similar between *rdm16ros1* and WT. These suggest that DNA methylation of short genes is largely dependent on the RdDM pathway and ROS1 demethylates the DNA methylation that is mainly contributed by RDM16 through the RdDM pathway. In the gene surrounding regions, mutation of *ROS1* caused increased DNA methylation levels of both 5′ promoters and 3′ downstream regions of the genes, with the methylation peaks close to gene ends (Figure 5B), which suggested that ROS1 might be important for the regulation of DNA methylation of gene regulatory elements and thus likely affects gene transcription. Mutation of *RDM16* in the *ros1* background reduced the CHH methylation to the wild-type level. Furthermore, RDM16 target loci were largely overlapping with ROS1 target loci. The active DNA demethylation machinery may recognize features resulting from RdDM and thus preferentially demethylate RdDM target sequences. It would be interesting to investigate whether RDM16 or its associated proteins may be involved in somehow marking the RdDM target sequences for demethylation.

## Materials and Methods

### Plant materials, growth conditions and mutant screening

The wild-type C24 and *ros1* mutant plants carry a homozygous RD29A promoter-driven luciferase transgene and a 35S promoter-driven *NPTII* transgene [41]. A T-DNA mutagenized *ros1* population was generated and screened for suppressors of *ros1* as described previously [43]. The *rdm16ros1* mutant was obtained from this screening. *rdm16-2* (CS861738), *rdm16-3* (CS861738) and another T-DNA insertion line (*SALK_057447C*) have the Columbia-0 (Col) genetic background. Plants were grown in a growth chamber or controlled room at 23°C with 16 h of light and 8 h of darkness.

### Cloning of *RDM16*

The *rdm16ros1* was crossed to *ros1* (C24 background) or *ros1-4* (Col background, Salk_045303) for genetic analysis or molecular mapping. The selfed F2 plants were subjected to the observation of luminescence emission and morphological alterations. For the mapping of *RDM16*, 30 F2 plants with low luminescence signal and defective morphology were used to determine the location of *RDM16* in the genome by using 25 indel polymorphic markers evenly distributed over the genome and then 74 F2 mutants and three more markers were used to map the *RDM16* on the chromosome 1. For the fine mapping of *RDM16*, a total of 632 F2 plants were used and 6 additional markers between At120 and At124 markers were developed. As a result, *RDM16* was finally mapped between At126 and At130 makers with a physical candidate region of 1.39 Mb. The second-generation high throughput DNA sequencing (Illumina) was carried out to search the mutation occurred in the *rdm16ros1* mutant. There was a DNA fragment inserted in the promoter of At1g28060. TAIL-PCR technique was used to determine the inserted position and the sequence of the DNA fragment [55].

For the complementation test of *rdm16ros1* mutant, a DNA fragment harboring 1.4 kb promoter, the gene and 0.7 kb downstream of stop codon of At1g28060 was amplified and finally cloned into a binary vector pORE-O2. The resultant vector was transformed into *rdm16ros1* mutant through Agrobacterium-mediated floral dip method. T2 plants were genotyped by genomic PCR and plants harboring the transgene were subjected for morphological observations, luminescence imaging and bisulfite sequencing analysis. For the luminescence imaging, leaves of 4-week-old plants were detached and treated with 2% NaCl for 3 h in the light at 23°C, and then applied for the luminescence imaging.

### Evaluation of the sensitivity to salt and ABA

For the sensitivity assay at germination stage, seeds of WT, *ros1* and *rdm16ros1* were sowed on a 1/2 MS plate containing 0, 75 mM NaCl or 0.5 µM ABA. The seed-containing plates were stratified at 4°C for 3 d and then exposed to a long day condition at 23°C. After 15 d treatment, the seedlings were photographed and compared. For the sensitivity assay at seedling stage, seeds were germinated and grown on a 1/2 MS plate for 6 d and then the seedlings were transferred to a 1/2 MS plate containing different concentrations of salt (0, 75, 100 or 125 mM NaCl) or ABA (0, 0.5, 1 or 2 µM ABA). After 7 d treatment, the root elongation was measured and compared.

### Expression analysis of mRNA, small RNA and Pol V transcripts

To examine the expression of *RD29A-LUC* and endogenous *RD29A*, 12-day-old seedlings were exposed to various stress conditions: 0, 150 mM NaCl for 3 h, 50 µM ABA for 3 h or cold (4°C) for 2 d. After the treatment, the whole seedlings were sampled for RNA isolation. For the expression analysis of *ROS1*, RdDM genes and Pol V transcripts, leaves of plants with three weeks old were collected for RNA isolation. Total RNA was extracted using the RNeasy Mini Kit (Qiagen) and then treated with Turbo DNase (Ambion) for 45 min to remove the contaminated DNA. Total RNA was used for first strand cDNA synthesis using a SuperScript II kit (Invitrogen), following the manufacturer's instructions with an oligo(dT)_12–18_ or random primer. The derived cDNA was used as template for semiquantitative or real-time RT-PCR analysis. For small RNA accumulation analysis, 2-week-old seedlings of the wild-type, *ros1*, *rdm16ros1*, *nrpd1ros1* and *nrpe1ros1* were harvested for the small RNA extraction. The extraction method and northern blot analysis of small RNA have been described previously [43]. The primers or probes for the expression analysis are listed in Table S9.

### DNA methylation analysis

Leaves of 4-week-old plants were collected for genomic DNA isolation. The DNA was extracted using the DNeasy Plant Mini Kit (Qiagen). To perform bisulfite sequencing, 80–100 ng of DNA was sodium-bisulfite converted and purified by using the BisulFlash DNA Modification Kit (EIPGENTEK). For the DNA methylation analysis, at least 15 clones at each sample were sequenced. The primers for the bisulfite sequencing are listed in Table S9.

### High-throughput RNA sequencing analysis

Leaves of the wild-type (Col) and *rdm16-2* plants with 4 weeks old were collected for RNA isolation. Total RNA was extracted using the RNeasy Mini Kit (Qiagen). The extracted RNA was sent to BGI (Shenzhen, China) for RNA-seq library preparation and whole transcriptome sequencing. The raw reads were aligned to the *Arabidopsis* genome (TAIR10, www.arabidopsis.org) by using TopHat program (http://tophat. cbcb.umd.edu). The assembling of the reads and the calculation of transcript abundance were performed by Cufflinks (http://cufflinks.cbcb.umd.edu). Transcripts that were differentially expressed in WT and *rdm16-2* were identified by Cuffdiff, a part of the Cufflinks package. For the intron-retention analysis, reads located in intron regions were calculated in WT and rdm16-2 separately, and then degrees of differential expression were measured according to the method described by Audic et al. (1997) [56], which was constructed based on Poisson distribution eliminating the influence of sequencing depth. The introns with more than 95% read coverage and false discovery rate (FDR) <0.01 were regarded as intron-retention events.”

### Whole-genome bisulfite sequencing and data analysis

DNA was extracted from leaves of 4-week-old plant and sent to BGI (Shenzhen, China) for bisulfite treatment, library preparation, and sequencing. For data analysis, adapter and low quality sequences (q<20) were trimmed and clean reads were mapped to a pseudo-C24 genome using BRAT-BW [57] allowing two mismatches. The pseudo-C24 genome was generated through the replacement of SNPs in the Col-0 genome with C24 variants (http://1001genomes.org/data/MPI/MPISchneeberger2011/releases/current//C24/Marker/C24.SNPs.TAIR9.txt). The method for identification of differentially methylated regions (DMRs) was according to Qian et al. (2012) with minor modifications [58]. In brief, differentially methylated cytosine (DMC) was identified if the p-value from the two-tailed Fisher's exact test was less than 0.05. DMRs were discovered using a sliding-window approach with 200 bp-window sliding at 50 bp intervals. A region with more than 2 DMCs was selected as an anchor region. The boundary of each anchor region was defined based on the locations of first and last DMCs in the region. If the distance between two anchor regions is less than 100 bp, they will be merged into one large region. DMRs were finally identified based on the regions with ≥100 bp, ≥5 DMCs, and absolute methylation difference of 0.3 for CG, 0.15 for CHG or 0.10 for CHH.

### ChIP assays

Twelve-day-old seedlings of C24 and *rdm16* mutant complementation lines with native promoter-driven RDM16-3xFLAG or RDM16-3xHA transgene were used for chromatin immunoprecipitation (ChIP) assays. The ChIP assays were performed according to a published protocol [59]. ChIP products were eluted into 50 µl of TE buffer, and a 2 µl aliquot was used for each qPCR reaction.

### Immunolocalization

Nuclei were isolated from protoplasts of *Arabidopsis* young leaves according to a published protocol [60]. Nuclei were then fixed in 4% formaldehyde and applied to slides for immunolocalization assay as previously described [61]. After being treated with the blocking solution (3% BSA in PBS), the nuclei were then incubated with primary antibodies overnight at 4°C. Each primary antibody was properly diluted in the blocking solution. After washing the slides, secondary anti-mouse TRITC (Invitrogen) and anti-rabbit FITC (Invitrogen) were added and incubated at 37°C. Chromatin was counterstained with DAPI. Images were acquired by SPINNING DISK confocal microscopy and then analyzed with Volocity software.

### Data deposition

The RNA-seq and whole-genome bisulfite sequencing data used in this paper have been deposited in the National Center for Biotechnology Information Gene Expression Omnibus (NCBI GEO) (http://www.ncbi.nlm.nih.gov/geo/) under accession numbers GSE44635 and GSE44417, respectively.

## Supporting Information

Figure S1 Sensitivity to kanamycin and DNA methylation level of *35S* promoter driving the expression of kanamycin resistance gene (*NPTII*). (**A**) Seeds of WT, *ros1* and *rdm16ros1* were germinated and grown on a plate containing 0, 25 or 50 mg/L kanamycin for 12 days. (**B**) DNA methylation analysis of *35S* promoter in WT, *ros1* and *rdm16ros1* through bisulfite sequencing.
(EPS)

Figure S2 Bisulfite sequencing of *SoloLTR* and *MEA-ISR* loci in WT, *ros1* and *rdm16ros1*. (**A**) *SoloLTR* loci, (**B**) *MEA-ISR* loci.
(EPS)

Figure S3 Molecular mapping of *RDM16*. (**A**) Primary mapping of *RDM16* using 72 F2 plants with reactivated LUC expression and morphological defects plants from *rdm16ros1*/*ros1-4* population. Physical distance and molecular markers are shown above vertical lines, and recombination rates between each marker and the gene are shown below the vertical lines. The long horizontal line represents the *Arabidopsis* chromosome 1 (**B**) Fine mapping of *RDM16* using 632 F2 mutants. The number of recombinants between each marker and the gene are shown below vertical lines. The *RDM16* gene was finally mapped between At126 and At130 markers, with a physical distance of 1.39 Mb.
(EPS)

Figure S4 Cloning of *RDM16*. (**A**) Genome sequencing of *rdm16ros1* and *ros1*. The gap in the promoter of At1g28060 was observed in *rdm16ros1* mutant. (**B**) The candidate mutation region could not be amplified in *rdm16ros1*. (**C**) DNA insertion and deletion occurred in the promoter of At1g28060 based on the results from TAIL-PCR analysis.
(EPS)

Figure S5 Expression level of At1g28060 was decreased in *rdm16ros1* mutant compared to *ros1* mutant. Real-time RT-PCR analysis was carried out to determine the expression of At1g28060 in *rdm16ros1* and *ros1* by using three primer pairs indicated in the figure for different regions of At1g28060.
(EPS)

Figure S6 Characterization of the T-DNA insertion line *rdm16-2*. (**A**) *rdm16-2* and *rdm16-3* have T-DNA inserted into the fifth intron and fifth exon of At1g28060, respectively. Primers for detection of the T-DNAs and the expression of At1g28060 were shown below the horizontal line. (**B**) Genomic genotyping of *rdm16-2* mutant. (**C**) Expression of the full At1g28060 cDNA in WT and *rdm16-2* by RT-PCR analysis. The full cDNA was not disrupted in the *rdm16-2* mutant. (**D**) Expression of At1g28060 gene in WT and *rdm16-2* mutant by real-time RT-PCR analysis. (**E**) Morphological alterations were occurred in *rdm16-2* mutant compared to WT. (**F**) DNA methylation analysis of *AtSN1* locus in WT and *rdm16-2* mutant. (**G**) Expression analysis of *ROS1* in WT, *rdm16-2*, *nrpd1* and *nrpe1*.
(EPS)

Figure S7 Domain structure of RDM16 and protein sequence alignment of RDM16 with its homolog. RDM16 has a conserved domain PRP3 and a DUF1115 domain with unknown function. The number indicates the position of amino acids of RDM16 protein. Os090249600, HPRP3 and Prp3 proteins are from rice, human being and yeast, respectively.
(EPS)

Figure S8 Mutation of *RDM16* did not affect the expression or pre-mRNA splicing of genes involved in the RdDM pathway. (**A**) Expression of genes encoding RdDM components in WT, *ros1* and *rdm16ros1* by real-time RT-PCR analysis. (**B**) RT-PCR analysis of genes encoding RdDM components in WT, *ros1* and *rdm16ros1*. Pre-mRNA splicing defects of genes encoding RdDM components were not observed in the *rdm16ros1* mutant.
(EPS)

Figure S9 Average CG and CHG methylation levels in the transposable elements (TEs) (**A**) and genes (**B**). TEs and genes were divided into 5 groups based on their size for detailed comparison of the DNA methylation levels among WT, *ros1*, *rdm16ros1*.
(EPS)

Figure S10 DNA methylation of 5 selected loci with decreased Pol V transcripts was altered in *rdm16ros1* compared to *ros1* control. (**A–E**) DNA methylation snapshot in IGV browser from the whole-genome bisulfite sequencing data. (**A**) RD29A promoter, (**B**) IGN5, (**C**) IGN28, (**D**) IGN30, (**E**) IGN32.
(EPS)

Figure S11 The *rdm16-2* mutation caused a reduction in Pol V transcripts. Quantitative RT-PCR analysis of Pol V transcripts in Col WT, *rdm16-2* and *nrpe1-11*.
(EPS)

Figure S12 Complementation test of *rdm16ros1* with RDM16-3xFlag or RDM16-3xHA construct. (**A**) Native promoter-driven RDM16-3xFlag or RDM16-3xHA rescued the morphological defects of *rdm16ros1* and restored the silencing of *RD29A-LUC* in *rdm16ros1*. (**B**) Western blot analysis of RDM16 protein in C24 WT, RDM16-3xFlag and RDM16-3xHA transgenic lines. The non-specific protein detected by Flag and HA antibodies served as an internal control.
(EPS)

Figure S13 Localization pattern of RDM16 in nuclei. Nuclei from C24 WT and RDM16-3xFlag transgenic plants were immunostained with anti-Flag and anti-U2B antibodies. The RDM16 protein was dispersed throughout the nucleoplasm without preferential accumulation in the Cajal body. U2B, a marker for the Cajal body.
(EPS)

Table S1 Intron-retention events occurred in *rdm16-2* mutant compared to WT.
(XLS)

Table S2 Genes upregulated in *rdm16-2* mutant compared to WT.
(XLS)

Table S3 Genes downregulated in *rdm16-2* mutant compared to WT.
(XLS)

Table S4 Bisulfite sequencing statistics.
(XLS)

Table S5 Hypomethylated loci in *rdm16ros1* compared to *ros1*.
(XLS)

Table S6 Hypermethylated loci in *rdm16ros1* compared to *ros1*.
(XLS)

Table S7 Hypomethylated loci in *nrpd1ros1* compared to *ros1*.
(XLS)

Table S8 Hypermethylated loci in *nrpd1ros1* compared to *ros1*.
(XLS)

Table S9 Primers or probes used in this study.
(XLS)
